# Genome-wide identification and analysis of the *EIN3/EIL* gene family in allotetraploid *Brassica napus* reveal its potential advantages during polyploidization

**DOI:** 10.1186/s12870-019-1716-z

**Published:** 2019-03-21

**Authors:** Mengdi Li, Ruihua Wang, Ziwei Liang, Xiaoming Wu, Jianbo Wang

**Affiliations:** 1 State Key Laboratory of Hybrid Rice, College of Life Sciences, Wuhan University, Wuhan, 430072 China; 2 Key Laboratory of Biology and Genetic Improvement of Oil Crops, Ministry of Agriculture, Oil Crops Research Institute of CAAS, Wuhan, 430062 China

**Keywords:** *EIN3/EIL* gene family, *Cis*-element, Introns, Allotetraploid, *Brassica napus*, Polyploidization

## Abstract

**Background:**

Polyploidization is a common event in the evolutionary history of angiosperms, and there will be some changes in the genomes of plants other than a simple genomic doubling after polyploidization. Allotetraploid *Brassica napus* and its diploid progenitors (*B. rapa* and *B. oleracea*) are a good group for studying the problems associated with polyploidization. On the other hand, the *EIN3/EIL* gene family is an important gene family in plants, all members of which are key genes in the ethylene signaling pathway. Until now, the *EIN3/EIL* gene family in *B. napus* and its diploid progenitors have been largely unknown, so it is necessary to comprehensively identify and analyze this gene family.

**Results:**

In this study, 13, 7 and 7 *EIN3/EIL* genes were identified in *B. napus* (2n = 4x = 38, A_n_C_n_), *B. rapa* (2n = 2x = 20, A_r_) and *B. oleracea* (2n = 2x = 18, C_o_). All of the identified EIN3/EIL proteins were divided into 3 clades and further divided into 8 sub-clades. Ka/Ks analysis showed that all identified *EIN3/EIL* genes underwent purifying selection after the duplication events. Moreover, gene structure analysis showed that some *EIN3/EIL* genes in *B. napus* acquired introns during polyploidization, and homolog expression bias analysis showed that *B. napus* was biased towards its diploid progenitor *B. rapa*. The promoters of the *EIN3/EIL* genes in *B. napus* contained more *cis*-acting elements, which were mainly involved in endosperm gene expression and light responsiveness, than its diploid progenitors. Thus, *B. napus* might have potential advantages in some biological aspects.

**Conclusions:**

The results indicated allotetraploid *B. napus* might have potential advantages in some biological aspects. Moreover, our results can increase the understanding of the evolution of the *EIN3/EIL* gene family in *B. napus*, and provided more reference for future research about polyploidization.

**Electronic supplementary material:**

The online version of this article (10.1186/s12870-019-1716-z) contains supplementary material, which is available to authorized users.

## Background

Polyploidization is widely recognized as an important mechanism for the formation of species in angiosperms [1–4]. Newly formed polyploids might experience rapid homolog loss, the alteration of gene expression patterns, genome restructuring post-polyploidization and other changes [5], which might vary greatly in different polyploids [6]. In addition, some gene families will also undergo changes during polyploidization, such as expansion of the gene family [7].

The *ethylene-insensitive 3 (EIN3)/ethylene-insensitive 3-like (EIL)* gene family is a small transcription factor gene family in higher plants [8, 9]. The *EIN3/EIL* genes participate in ethylene signal transduction by activating downstream ethylene response genes [10, 11]. Ethylene (ET), an important gaseous plant hormone, is involved in some important physiological processes that regulate the growth, development and senescence of plants [12]. In addition, ethylene can act as a signal molecule to regulate the expression of some genes [13]. Thus, the *EIN3/EIL* gene family plays an important role in plants. EIN3/EIL proteins are characterized by two structural features. One feature is that their N-terminal amino acid sequences are highly conserved with several significant structural features [14] and these sequences, except for the first ~ 80 amino acid residues, are also essential for the activity of proteins [15, 16]. The other feature is that their C-terminal sequences are less conserved than their N-terminal sequences. For instance, in some plants, such as *Arabidopsis thaliana* [14] and mung bean [13], the poly-asparagine or poly-glutamine region widely exists in the C-terminal sequences of EIN3/EIL proteins, but such features are not found in other plants, such as tobacco [17].

The functions and characteristics of some *EIN3/EIL* genes have been well studied in several plants, such as *A. thaliana* and tobacco. EIN3 regulates the expression of its downstream gene ETHYLENE-RESPONSE-FACTOR1 (ERF1) and it is also involved in the transcriptional regulation initiated by ethylene in Arabidopsis [14, 15]. EIL3 (or SLIM1) functions as the central transcriptional regulator of sulfur response and metabolism in Arabidopsis [18, 19]. NtEIL2, the homolog of AtSLIM1, directly regulates the expression of some genes induced by sulfur starvation by binding to the UP9C promoter in tabacco [20]. All of the above findings have shown that EIN3/EIL proteins have a complex relationship with the ethylene and sulfur signaling pathways.

*Brassica napus*, a typical allotetraploid of the *Brassica* genus, is the third largest oil crop planted worldwide. *B. napus* (2n = 4x = 38, A_n_C_n_) was formed ~ 7500 years ago by natural hybridization and polyploidization of *B. rapa* (2n = 20, A_r_) and *B. oleracea* (2n = 18, C_o_). In recent years, the whole genomes of *B. rapa* (Chiifu-401-42), *B. oleracea* (*capitata*-02-12) and *B. napus* (Darmor-*bzh*) have been sequenced and assembled [21–23]. The purpose of this study was to improve our understanding of the *EIN3/EIL* gene family in allotetraploid *B. napus* and to explore the changes in this gene family during the formation of *B. napus*. Some methods were used in this study, including gene structure analysis, chromosomal localization analysis, phylogenetic trees analysis, synteny and duplicated genes analysis, promoter analysis and expression profiles analysis.

## Results

### Identification and chromosomal localization of *EIN3/EIL* genes

The BLASTp program in the BRAD database was used to identify *EIN3/EIL* genes in *B. napus* and its diploid progenitors (*B. rapa* and *B. oleracea*), and the query sequences were six EIN3/EIL protein sequences in *A. thaliana* from TAIR database. All alternative protein sequences were confirmed by CD-search in NCBI, and the domain ID was pfam04873. Finally, 7, 7 and 13 genes were identified as *EIN3/EIL* genes in *B. rapa, B. oleracea* and *B. napus*, respectively. These identified *EIN3/EIL* genes were named from *BrEIL1* to *BrEIL4b* in *B. rapa*, *BoEIL1* to *BoEIL4b* in *B. oleracea* and *BnCEIN3* to *BnAEIL4d* in *B. napus* (Table 1). The last letter in these names represented the homologous relationship with the *EIN3/EIL* genes in *A. thaliana*, with ‘*a*’ meaning the highest homology, followed by ‘*b*’ and so on. The letters *A* and *C,* following ‘*Bn*’, represented the A_n_ and C_n_ sub-genomes, respectively, in gene names of *B. napus*.

**Table 1:** The information of *EIN3/EIL*s in *B. napus* and its diploid progenitors with their Arabidopsis orthologs

Gene name	BRAD ID	Chromosome			Strand	Block	CDs (bp)	Orthologous gene
		No.	Start	End				
BrEIL1	Bra000528	A03	11,612,582	11,614,297	+	I	1716	AT2G27050
BrEIL2	Bra002358	A10	10,160,414	10,161,946	–	R	1533	AT5G21120
BrEIL3a	Bra015970	A07	23,293,714	23,295,723	–	E	1764	AT1G73730
BrEIL3b	Bra003831	A07	18,534,952	18,536,782	+	E	1659	AT1G73730
BrEIL3c	Bra008110	A02	12,192,159	12,193,778	–	E	1620	AT1G73730
BrEIL4a	Bra028601	A02	1,519,950	1,521,317	+	R	1368	AT5G10120
BrEIL4b	Bra006050	A03	1,762,948	1,764,210	+	R	990	AT5G10120
BoEIL1	Bol032895	C06	5,998,916	6,000,631	+	I	1716	AT2G27050
BoEIL2a	Bol036088	C02	5,947,506	5,949,050	+	R	1545	AT5G21120
BoEIL2b	Bol035762	C09	29,569,796	29,571,388	–	R	1593	AT5G21120
BoEIL3a	Bol026238	C06	3,515,537	3,517,637	+	E	1758	AT1G73730
BoEIL3b	Bol039991	C06	33,246,791	33,248,491	+	E	1701	AT1G73730
BoEIL4a	Bol024640	C02	2,716,756	2,718,117	+	R	1362	AT5G10120
BoEIL4b	Bol008759	C03	1,888,488	1,889,829	+	R	1074	AT5G10120
BnCEIN3	BnaC01g32460D	chrC01	31,744,455	31,747,102	+	F	1764	AT3G20770
BnCEIL1a	BnaC03g26570D	chrC03	15,142,985	15,145,564	+	I	1692	AT2G27050
BnAEIL1b	BnaA03g22560D	chrA03	10,717,296	10,719,627	+	I	1713	AT2G27050
BnAEIL2a	BnaA10g14470D	chrA10	11,504,970	11,506,502	–	R	1500	AT5G21120
BnCEIL2b	BnaCnng35080D	chrCnn_random	33,278,634	33,280,328	–	R	1530	AT5G21120
BnAEIL3a	BnaA07g39030D	chrA07_random	1,960,115	1,962,709	–	E	1764	AT1G73730
BnCEIL3b	BnaC06g34570D	chrC06	33,988,588	33,991,257	–	E	1758	AT1G73730
BnCEIL3c	BnaC06g23450D	chrC06	25,297,162	25,299,268	+	E	1698	AT1G73730
BnAEIL3d	BnaA02g16550D	chrA02	9,876,349	9,880,706	–	E	1749	AT1G73730
BnCEIL4a	BnaC02g00520D	chrC02	216,092	217,453	–	R	1362	AT5G10120
BnAEIL4b	BnaA02g00350D	chrA02	129,692	131,059	+	R	1368	AT5G10120
BnCEIL4c	BnaC03g04150D	chrC03	1,999,482	2,000,328	+	R	579	AT5G10120
BnAEIL4d	BnaA03g02810D	chrA03	1,359,358	1,360,620	+	R	990	AT5G10120

**Fig. 1:**
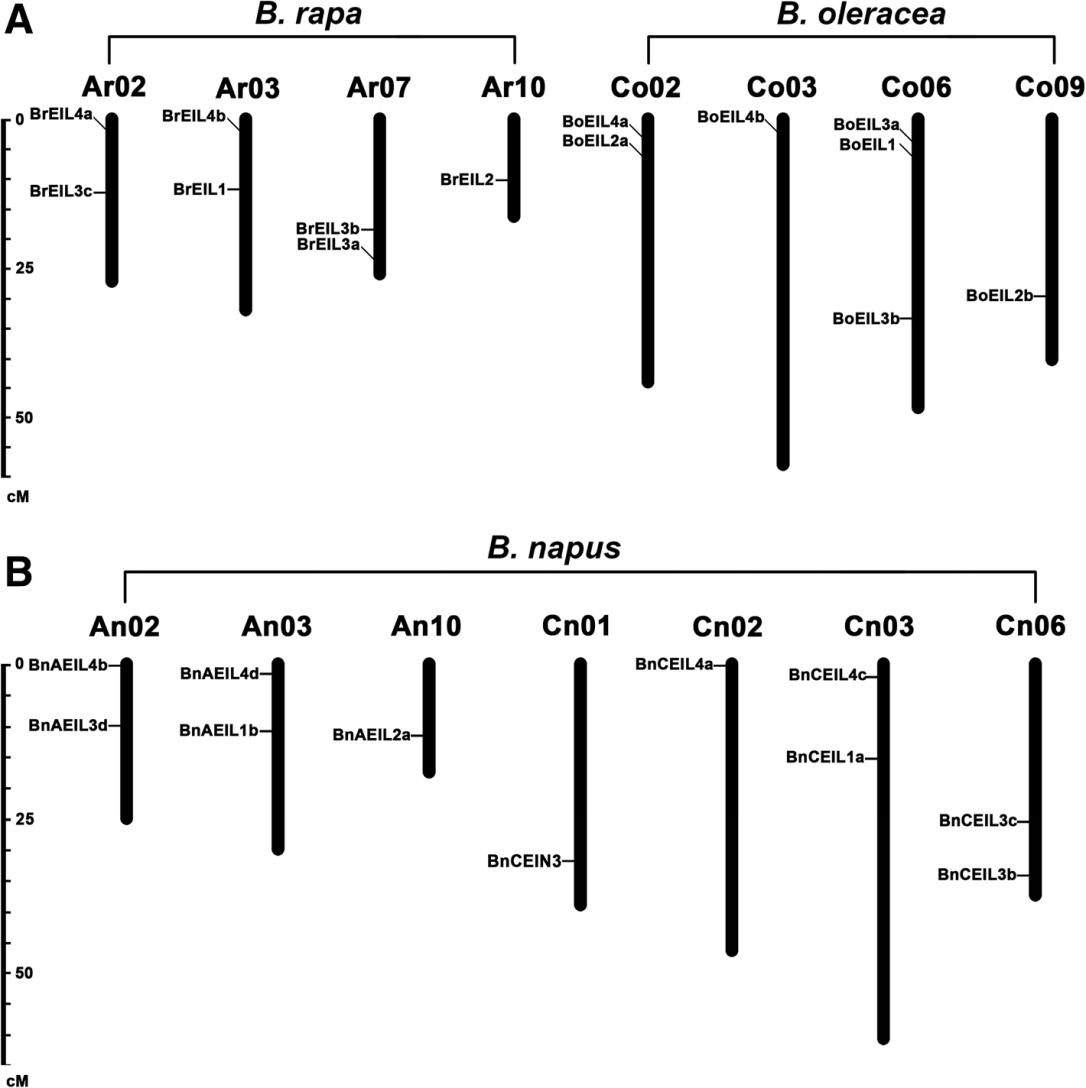
Chromosome distribution of *EIN3/EILs* in *B. rapa, B. oleracea* (**a**) and *B. napus* (**b**). Genes located in unassembled scaffolds were not shown in this figure. The number of chromosomes was marked at the top of each chromosome, and the scale on the left is in megabases (Mb)

The physical locations of identified *EIN3/EIL* genes in *B. napus* and its two diploid progenitors were drafted to corresponding chromosomes by the MapInspector tool. Twenty-five out of twenty-seven *EIN3/EIL* genes could be mapped to assembled chromosomes (Fig. 1), and the other two genes (*BnCEIL2b* & *BnAEIL3a*) were located on unassembled scaffolds. Seven *EIN3/EIL* genes were located on four chromosomes (A_r_02, A_r_03, A_r_07 and A_r_10) in the A genome in *B. rapa*, only five genes were on three chromosomes (A_n_02, A_n_03 and A_n_10) in the A sub-genome in *B. napus*. Comparing the gene distribution of the A sub-genome in *B. napus* with the A genome in *B. rapa*, the genes on the corresponding chromosomes not only were homologous genes, but they also had the same relative positions, except for the A_r_07 chromosomes, two *EIN3/EIL* genes on which might have been lost during the formation of *B. napus* or due to incomplete assembly of this chromosomes. Moreover, comparing the gene distribution of the C sub-genome in *B. napus* with the C genome in *B. oleracea*, only a few *EIN3/EIL* genes maintained their relative positions on the corresponding chromosomes. In addition, a total of 8 homologous gene pairs (such as *BrEIL4a* & *BnAEIL4b*, *BrEIL3c* & *BnAEIL3d*) maintained their relative position on chromosomes during the formation of *B. napus*. Therefore, the A sub-genome of *B. napus* might be more stable than the C sub-genome during the process of hybridization and polyploidization.

### Phylogenetic analysis of EIN3/EIL proteins

**Fig. 2:**
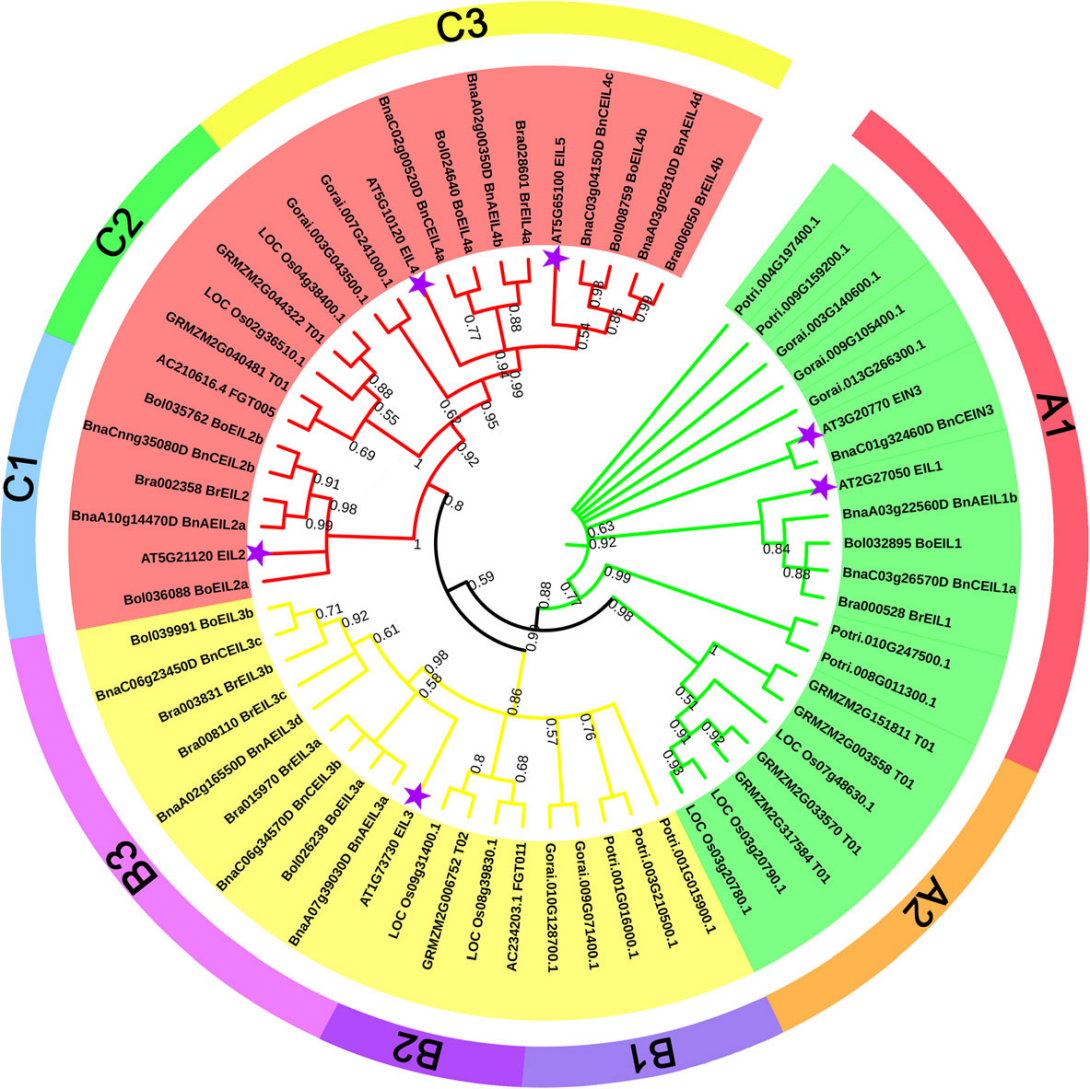
Phylogenetic tree of EIN3/EIL proteins in 8 species. The tree was constructed using MEGA7.0 with the Maximum Likelihood (ML) method and 1000 bootstrap replicates. The prefixes Bra, Bol, Bna, Potri, AT, LOC, GRMZM/AC, and Gorai stand for *B. rapa*, *B. oleracea*, *B. napus*, *Populus trichocarp,* Arabidopsis, *Oryza sativa, Zea mays* and *Gossypium raimondii*, respectively. The inner circle is marked in green, yellow and red representing the Clade A, Clade B, and Clade C, respectively. Each clade was divided into sub-clades, and marked in different colors on the outer circle. Only bootstrap values greater than 50% were displayed. The purple stars represented the *EIN3/EIL* genes in Arabidopsis

A total of 63 EIN3/EIL protein sequences from 8 different species were used as reference sequences to construct the phylogenetic tree, including 6 (Arabidopsis), 7 (*B. rapa*), 7 (*B. oleracea*), 13 (*B. napus*), 7 (*Oryza sativa*), 7 (*Populus trichocarpa*), 7 (*Gossypium raimondii*) and 9 (*Zea mays*) members (Fig. 2). These 63 EIN3/EIL proteins were obviously divided into three clades, designated as A, B and C, which contained 8 sub-clades (A1, A2, B1, B2, B3, C1, C2 and C3). Clade A contained EIN3 and EIL1 proteins, clade B contained EIL3 proteins, and clade C consisted of EIL2, EIL4 and EIL5 proteins. The EIN3/EIL proteins in monocots and dicots were clustered in different sub-clades in this phylogenetic tree, e.g., EIN3/EIL proteins in monocots (*Zea mays* and *Oryza sativa*) were clustered in the A2, B2 and C1 sub-clades, while EIN3/EIL proteins in dicots were clustered in the remaining sub-clades. Clade A had 21 EIN3/EIL proteins, clade B had 19 proteins, and clade C had 23 proteins, so the *EIN3/EIL* genes were evenly classified into three clades.

### Gene structure analysis of *EIN3/EIL* genes

**Fig. 3:**
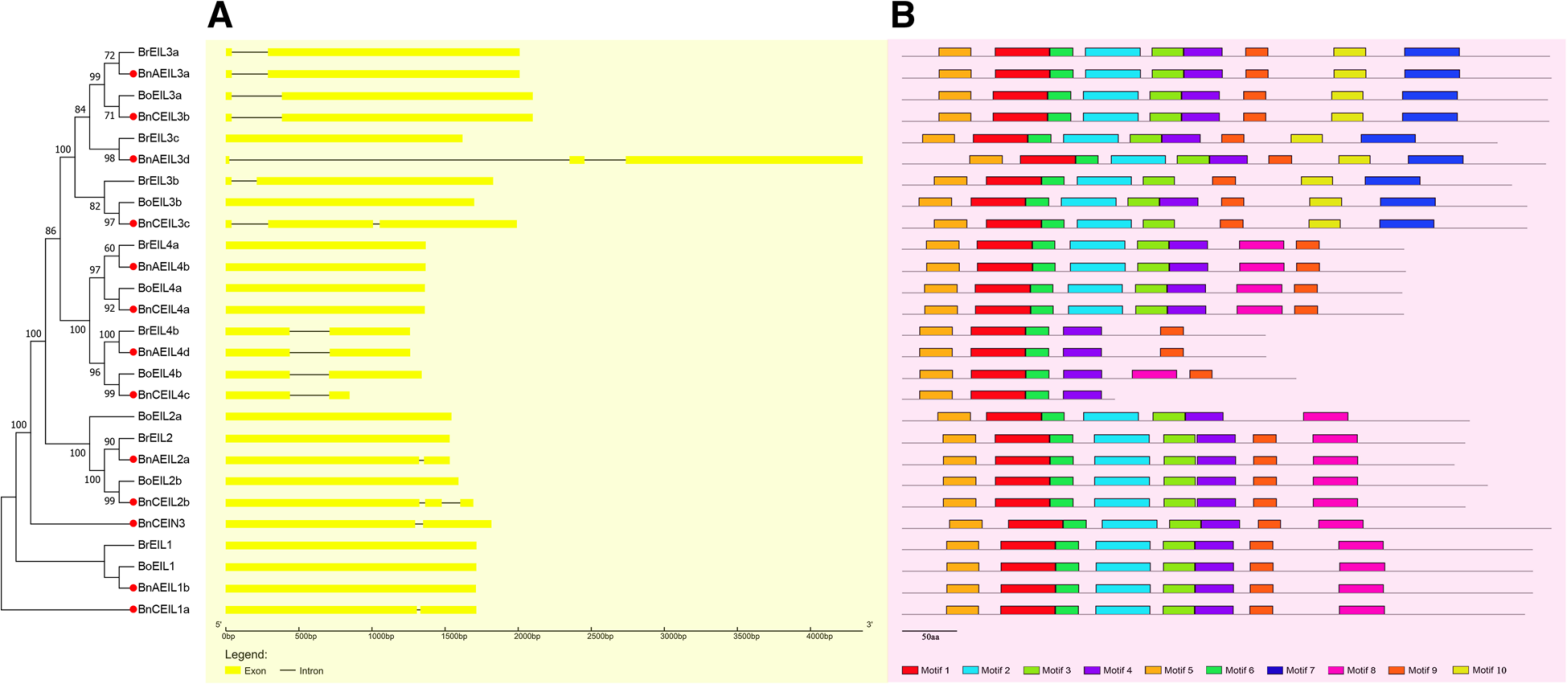
Characterizations of the identified *EIN3/EIL* genes, including gene structure **(a)** and conserved motif location **(b)**. The *EIN3/EIL* genes in allotetraploid *B. napus* were marked by the red circle

The diversity of gene structure is the main resource for the evolution of multigene families [24–26]. To explore the structural diversity of identified *EIN3/EIL* genes, the exon-intron structure of these genes was analyzed. As shown in Fig. 3a, twelve *EIN3/EIL* genes did not contain any introns, such as *BrEIL3c*, *BoEIL3b*, and *BnAEIL4b*. Twelve genes contained only one intron, such as *BrEIL3a*, *BoEIL3a* and *BnCEIL3b*, whereas the remaining three genes (*BnAEIL3d*, *BnCEIL3c* and *BnCEIL2b*) contained two introns. Since all genes containing two introns were genes in the allotetraploid *B. napus*, it was speculated that some members of the *EIN3/EIL* gene family acquired additional introns during the polyploidization process. To further analyze whether the gene structure of *EIN3/EIL* genes has altered during the process of polyploidization, 11 pairs of genes (Table 2) with the closest genetic distance were selected for further comparative analysis. Among these genes, four pairs of genes had different gene structures, and three of them had acquired two introns in the *EIN3/EIL* genes of the allotetraploid, such as *BnAEIL3d*, *BnCEIL3c* and *BnCEIL2b*, while the other pair of genes had obtained one intron (*BnAEIL2a*). In addition, although a pair of genes (*BoEIL4b* & *BnCEIL4c*) had an identical gene structure, *BnCEIL4c* apparently lost part of its second exon compared to the corresponding gene in diploid *B. oleracea*. These results suggest that intron/exon acquisition or loss events have happened during the evolution of the *EIN3/EIL* gene family in *B. napus*, which might explain the functional divergence of the homologous *EIN3/EIL* genes.

**Table 2:** Information about *EIN3/EIL* gene pairs with potential direct evolutionary relationships

Clade	*EIN3/EIL* genes in diploid progenitors		*EIN3/EIL* genes in allotetraploid *B. napus*	
	Gene name	The number of exons	Gene name	The number of exons
A1 sub-clade	BoEIL1	1	BnAEIL1b	1
B3 sub-clade	BrEIL3a	2	BnAEIL3a	2
	BoEIL3a	2	BnCEIL3b	2
	BrEIL3c	1	BnAEIL3d	3
	BoEIL3b	1	BnCEIL3c	3
C1 sub-clade	BrEIL2	1	BnAEIL2a	2
	BoEIL2b	1	BnCEIL2b	3
C3 sub-clade	BrEIL4a	1	BnAEIL4b	1
	BoEIL4a	1	BnCEIL4a	1
	BrEIL4b	2	BnAEIL4d	2
	BoEIL4b	2	BnCEIL4c	2

Next, the MEME server was used to find some conserved motifs in EIN3/EIL proteins (Fig. 3b). As a result, ten most conserved motifs were identified, in which motifs 1, 5 and 6 were present in all EIN3/EIL proteins. According to a previous study, motifs 1, 6, 3 and 4 might constitute a conserved domain of EIN3/EILs [27]. Remarkably, all proteins in clade B contained a unique motif 7, suggesting that this motif might have a special function that distinguished the function of these proteins from other EIN3/EIL proteins. Moreover, most of the closely related EIN3/EILs exhibited similar motif compositions, such as BrEIL3a & BnAEIL3a, BrEIL4a & BnAEIL4b, and BrEIL2 & BnAEIL2a, indicating the functions between them might be extremely similar.

### Conserved amino acid and characteristic analysis of EIN3/EIL proteins

**Fig. 4:**
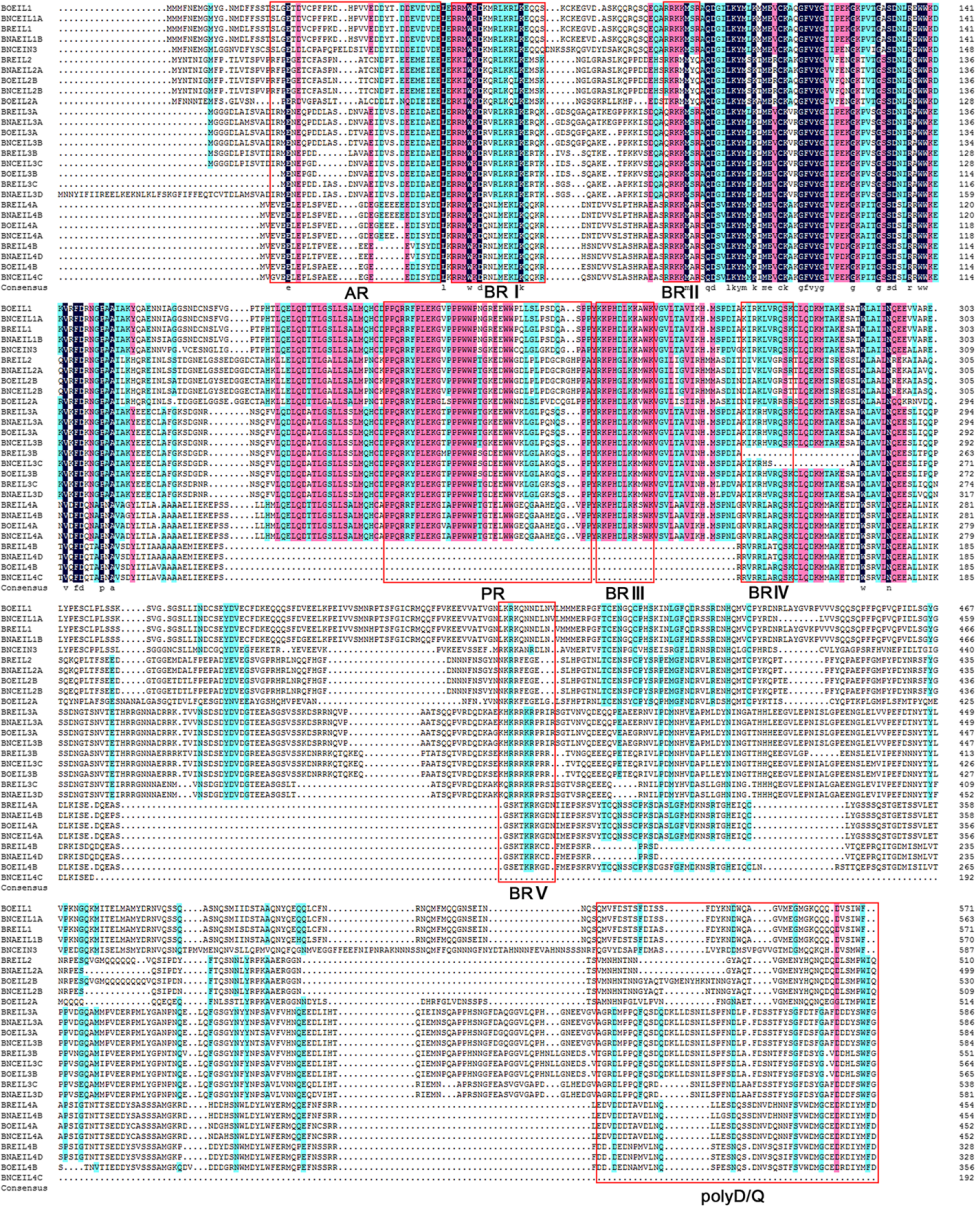
Sequence alignment of all identified *EIN3/EIL* genes. Sequences were aligned by ClustalX, and identical or similar residues were shaded as colors. Red rectangle covers the structural features. AR: acidic region; BRI-V: basic region I-V; PR: proline-rich region; ploy Q/D: poly Asp/Gln region

To further evaluate the identity of the EIN3/EIL protein sequences of *B. napus* and its diploid progenitors, all sequences were aligned together and similar or identical residues were shaded in different colors (Fig. 4). Different from the highly conservative N-terminal sequences of the EIN3/EIL proteins, C-terminal sequences did not show significant similarity, suggesting that these sequences were the major sources of the variations of EIN3/EIL members. The N-terminal sequences of all EIN3/EIL proteins in Arabidopsis exhibit some structural features, such as acidic N-terminal amino acids, basic amino acid clusters and a proline-rich domain [14]. In this study, four structural features were identified and refined in the EIN3/EIL proteins of *B. napus* and its diploid progenitors: 1) a highly acidic region (AR) at the N-terminus; 2) five conserved basic regions (BRI-V); 3) a proline-rich region (PR); and 4) a poly-Asp/Gln (Q/D) region (Fig. 4). In detail, the N-terminal AR mainly includes many Asp (D) and Glu (E) residues. Five BRs, including Arg (R), Lys (K) and His (H) residues, were scattered in the first part of the EIN3/EIL proteins. And the Pro (P) residue in the PR was very conserved except in four proteins (BrEIL4b, BnAEIL4d, BoEIL4b and BnCEIL4c). Regions rich in acidic amino acids, proline and glutamine are common transcriptional activation domains in some plants [14, 28]. Therefore, the amino acid composition of the first half of the EIN3/EIL proteins in *B. napus* and its diploid progenitors demonstrated their roles in transcriptional activation.

As shown in Table 3, the length of identified EIN3/EIL proteins ranged from 192 (BnCEIL4c) to 587 (BnCEIN3 & BnAEIL3a) amino acids in *B. napus*. Additionally, the physical and chemical properties of all 27 EIN3/EIL proteins were analyzed online (Table 3), including molecular weight (MW), theoretical pI, instability index (II), aliphatic index and grand average of hydropathicity (GRAVY). The predicted MWs were between 22.17 kDa (BnCEIL4c) and 66.60 kDa (BnCEIN3) in *B. napus*. By calculation, the average number of amino acids in *B. napus* (498) was lower than that in its diploid progenitors (509), and the MW in *B. napus* (56.58 kDa) was also lower than that in its diploid progenitors (57.76 kDa). This indicated that members of the *EIN3/EIL* gene family might have lost partial amino acid sequences in *B. napus* during the process of polyploidization. All EIN3/EIL proteins in *B. napus* and its diploid progenitors had an instability index greater than 40, indicating that they were all unstable proteins. All EIN3/EIL proteins were confirmed as hydrophilic proteins with the negative GRAVY values. Among all EIN3/EIL proteins, the shortest domain was 155 amino acids, such as in BnCEIL4c, BnAEIL4d, and the longest was 584 amino acids, such as in BrEIL3a and BnAEIL3a. The tertiary structure of all EIN3/EIL proteins in *B. napus* and its diploid progenitors was predicted using the homology modeling method (SWISS-MODEL) (Additional file 1: Figure S1). Results showed that the EIN3/EIL proteins mainly matched two templates. One was the three-dimensional structure of the DNA-binding domain (DBD) of AtEIN3 protein (SMTL ID: 4zds.1), which is composed of six α-helices and five short helical turns [29]. The other was the three-dimensional structure of the DBD of AtEIL3 (SMTL ID: 1wij.1), which is composed of 5 α-helices [8].

**Table 3:** The predicated protein information of EIN3/EILs in *B. napus* and its diploid progenitors

Gene name	No. of amino acids	Domin location	Mol. Wt (kDa)	Isoelectric point (pI)	Instability index (II)	Aliphatic index	Grand average of hydropathicity (GRAVY)
BrEIL1	571	48–297	65.06	5.8	56.57	62.61	−0.714
BrEIL2	510	49–299	58.07	6.88	53.57	53.55	−0.93
BrEIL3a	587	4–587	65.97	5.16	62.17	65.6	−0.861
BrEIL3b	552	4–552	62.53	5.29	60.82	66.9	−0.878
BrEIL3c	539	1–539	60.80	5.43	58.6	67.12	−0.815
BrEIL4a	455	30–274	51.77	4.99	61.1	66.24	−0.82
BrEIL4b	329	24–178	38.10	4.97	70.26	58.66	−0.903
BoEIL1	571	48–297	65.27	5.96	56.26	63.29	−0.696
BoEIL2a	514	40–288	58.33	6.34	50.28	65.06	−0.788
BoEIL2b	530	49–299	60.42	6.27	53.55	52.81	−0.964
BoEIL3a	585	4–585	65.73	5.2	62.25	64.82	−0.869
BoEIL3b	566	1–566	64.23	5.39	64.92	65.62	−0.924
BoEIL4a	453	28–272	51.37	5.02	56.92	66.31	−0.787
BoEIL4b	357	24–178	41.05	4.88	73.27	58.43	−0.89
BnCEIN3	587	51–303	66.60	5.38	48.8	62.4	−0.726
BnCEIL1a	563	48–297	64.38	5.73	56.39	62.98	−0.702
BnAEIL1b	570	48–297	64.98	5.92	54.21	62.72	−0.716
BnAEIL2a	499	49–299	56.78	6.88	53.22	53.95	−0.911
BnCEIL2b	509	49–299	57.92	6.22	52.47	53.28	−0.942
BnAEIL3a	587	4–587	66.04	5.16	61.39	64.28	−0.874
BnCEIL3b	585	4–585	65.73	5.2	62.25	64.82	−0.869
BnCEIL3c	565	4–565	63.90	5.25	62.55	66.94	−0.889
BnAEIL3d	582	34–582	65.87	5.46	55.32	69.19	−0.744
BnCEIL4a	453	28–272	51.41	4.98	58.22	66.11	−0.786
BnAEIL4b	455	30–274	51.72	5.02	61.53	66.02	−0.821
BnCEIL4c	192	24–178	22.17	8.75	79.13	76.72	−0.769
BnAEIL4d	329	24–178	38.10	4.97	70.26	58.66	−0.903

### Synteny and duplicated gene analysis of *EIN3/EIL* genes

**Fig. 5:**
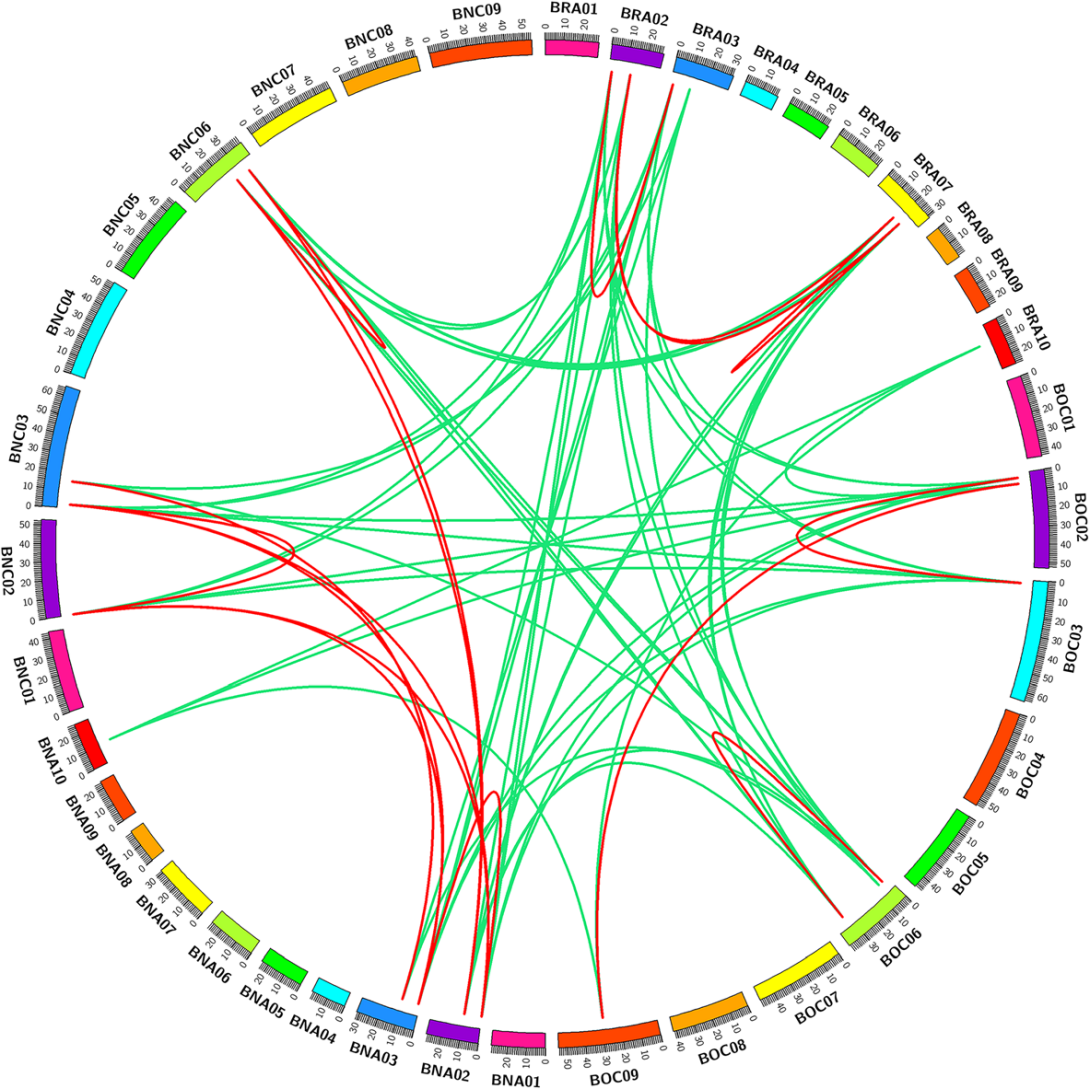
Genome-wide synteny analysis for *EIN3/EIL* genes among *B. napus* and its diploid progenitors. BRA01–10 and BOC01–09 represented chromosomes in *B. rapa* and *B. oleracea*, respectively. BNA01–10 and BNC01–09 represented chromosomes in the A_n_ and C_n_ sub-genomes in *B. napus*, respectively. All identified *EIN3/EIL* genes were mapped onto corresponding chromosomes. Green lines linked the syntenic orthologs and red lines linked the syntenic paralogs

The synteny relationship of *EIN3/EIL* genes was analyzed using the genome information from *B. napus* (A_n_ and C_n_) and its diploid progenitors (A_r_ and C_o_) with the syntenic information from the BRAD database. A total of 17 pairs of *EIN3/EIL* syntenic paralogs and 51 pairs of syntenic orthologs were found in these genomes (Fig. 5). Ten pairs of syntenic paralogs were observed in *B. napus*, and each pair of syntenic *EIN3/EIL* genes corresponded to a homologous gene in Arabidopsis. For example, a pair of syntenic paralogs (*BnCEIL1a* and *BnAEIL1b*) were located on C_n_03 and A_n_03 chromosomes, respectively, and all of them exhibited high sequence similarities with *AtEIL1* (AT2G27050). Moreover, compared with the number of syntenic orthologous *EIN3/EIL* genes of *B. rapa* and *B. oleracea*, 20 orthologous genes were observed between *B. rapa* and *B. napus*, and 18 between *B. oleracea* and *B. napus*. In addition, 13 pairs of syntenic orthologous genes were found in *B. rapa* and *B. oleracea*, only 7 pairs were found in the two sub-genomes of *B. napus*, indicating that some of the syntenic *EIN3/EIL* genes might be lost during the process of polyploidization.

Most duplicated genes have been silenced for millions of years, with only a few surviving and further undergoing intense purifying selection after duplication events [30]. To obtain more insight into whether selective pressure was associated with the *EIN3/EIL* genes after duplication events, the non-synonymous (Ka) and synonymous substitution (Ks) values were calculated for the 10 identified duplicated gene pairs (Table 4). According to the ratio of Ka and Ks, the selection pressure for duplicated genes can be presumed. The value of Ka/Ks = 1 indicates that genes were undergoing neutral selection, Ka/Ks > 1 means that genes were selected positively, and Ka/Ks < 1 shows that genes undergoing purifying selection [31]. As shown in Table 4, the Ka/Ks values from all 10 gene pairs were less than 1, indicating that the *EIN3/EIL* gene family in *B. napus* and its diploid progenitors has undergone purifying selection pressure after the duplication events.

**Table 4:** The Ka and Ks values of duplicated *EIN3/EIL* gene pairs

Duplicated gene pairs	Ks	Ka	Ka/Ks	Duplication type	Types of selection
BrEIL3a-BrEIL3c	0.449	0.082	0.183	Segmental	Purify selection
BrEIL3a-BrEIL3b	0.397	0.076	0.192	Segmental	Purify selection
BoEIL3a-BoEIL3b	0.435	0.071	0.163	Segmental	Purify selection
BnCEIL1a-BnAEIL1b	0.072	0.015	0.215	Segmental	Purify selection
BnAEIL2a-BnCEIL2b	0.074	0.019	0.260	Segmental	Purify selection
BnAEIL3a-BnCEIL3b	0.118	0.015	0.130	Segmental	Purify selection
BnAEIL3a-BnCEIL3c	0.396	0.077	0.193	Segmental	Purify selection
BnCEIL3b-BnCEIL3c	0.441	0.076	0.173	Segmental	Purify selection
BnCEIL3c-BnAEIL3d	0.441	0.102	0.232	Segmental	Purify selection
BnCEIL4a-BnAEIL4b	0.137	0.012	0.089	Segmental	Purify selection

### Analysis of *cis-*acting elements in the promoters of *EIN3/EIL* genes

**Fig. 6:**
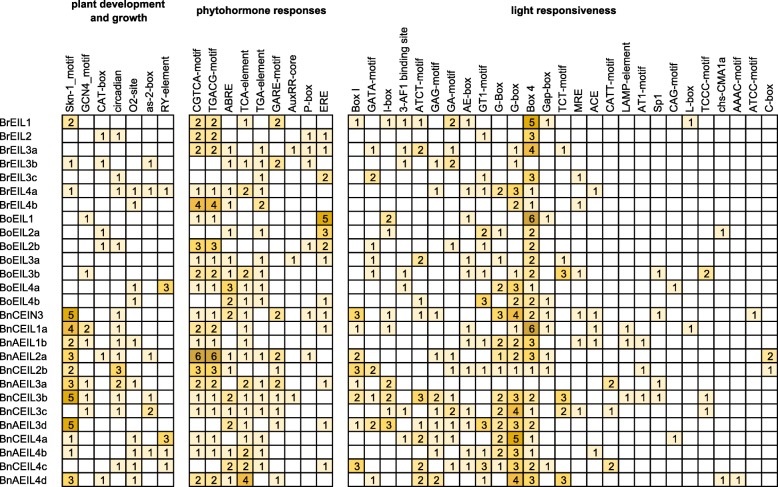
*Cis*-acting elements on promoters of all identified *EIN3/EIL* genes

The presence of different *cis-*acting elements in promoters of genes might imply that the functions of these genes were different. To explore the *cis*-acting elements in the promoters of *EIN3/EIL* genes, a 1.5 kb genomic sequence upstream of the transcription start site (TSS) in each gene was extracted and then searched in the PlantCARE database [32]. As shown in Fig. 6, the *cis*-acting elements responsible for plant development and growth, phytohormone responses and light responsiveness in the promoters of all *EIN3/EIL* genes in *B. napus* and its two diploid progenitors were identified and counted. Seven *cis*-acting elements were associated with plant development and growth and two of them (Skn-1_motif and GCN4_motif) [33] were involved in endosperm gene expression. Most (84.6%) promoters of *EIN3/EIL* genes contained a Skn-1_motif in the allotetraploid *B. napus*, while few promoters of *EIN3/EIL* genes contained Skn-1_motif in the diploid *B. rapa* and *B. oleracea*. CAT-box [34], a *cis*-acting regulatory element related to meristem expression, were found in some *EIN3/EIL* genes in *B. napus* and its two diploid progenitors. The circadian control element, circadian [35], was also found in many promoters of *EIN3/EIL* genes in *B. napus*, such as *BnCEIL2b* and *BnAEIL3a*. The remaining *cis*-acting elements associated with plant development and growth were zein metabolism regulatory elements (O2-site) and the as-2-box and RY-element [36], which are specific for shoot and seed development.

For phytohormone response-related *cis*-acting regulatory elements, CGTCA-motif and TGACG-motif [37] involved in the MeJA-responsiveness were identified at the *EIN3/EIL* gene promoters in *B. napus* and its two diploid progenitors. Auxin-responsive elements (TGA-element and AuxRR-core) [38] and gibberellin-responsive elements (GARE-motif and P-box) [39] were also found in some *EIN3/EIL* gene promoters. ABRE [40] and TCA-element, which are related to the abscisic acid and salicylic acid responsiveness, respectively, were found in most *EIN3/EIL* gene promoters. ERE, an ethylene-responsive element, was also present in some *EIN3/EIL* gene promoters. Moreover, *BoEIL1* had the largest number (5) of EREs in its promoter. A total of 27 elements were associated with light responsiveness in the promoters of all identified *EIN3/EIL* genes, such as Box 4, G-box and GT1-motif. It was worth noting that most of the *cis*-regulatory elements observed in the identified *EIN3/EIL* gene promoters in *B. napus* and its two diploid progenitors were primarily associated with light responsiveness.

### Gene expression pattern analysis of *EIN3/EIL* genes

**Fig. 7:**
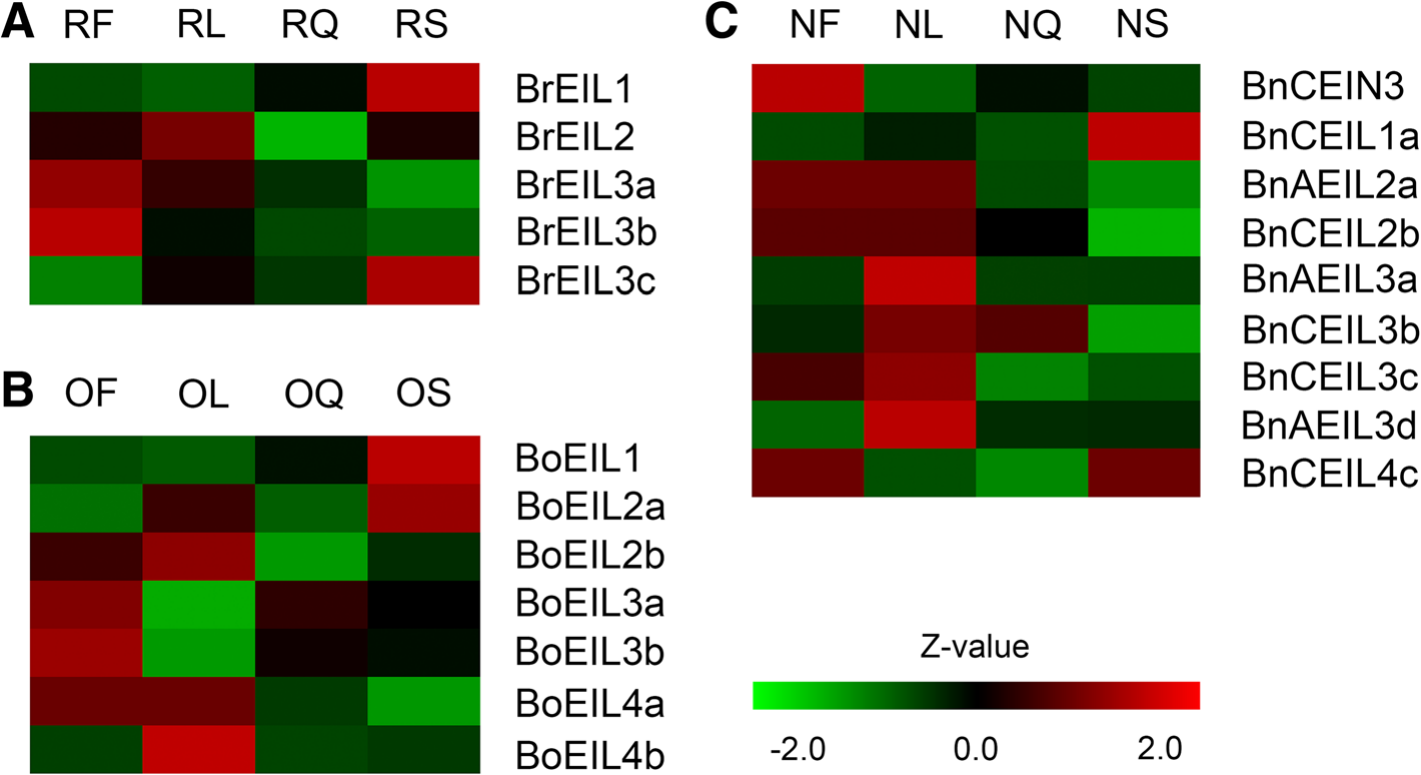
Expression patterns of identified *EIN3/EILs* in stems, leaves, flowers and siliques. **a** The expression patterns of *EIN3/EILs* in *B. rapa*. **b** The expression patterns of *EIN3/EILs* in *B. oleracea*. **c** The expression patterns of *EIN3/EILs* in *B. napus*

To further understand the expression of all identified *EIN3/EIL* genes and their potential biological functions, their expression patterns in four major tissues (leaves, stems, flowers and siliques) were investigated based on our RNA-seq data (Additional file 2: Table S1). Overall, the expression of all *EIN3/EIL* genes were not tissue-specific in these four tissues, indicating that they might play roles in all these tissues (Fig. 7). As shown in Additional file 2: Table S1, a total of 6 *EIN3/EIL* genes were not expressed in selected tissues. Among them, *BrEIL4a* and *BrEIL4b* were not expressed in all four tissues in *B. rapa*, and 4 genes (*BnAEIL1b*, *BnCEIL4a*, *BnAEIL4b* and *BnAEIL4d*) were not expressed in *B. napus*. As seen in Fig. 7, homologous genes of *EIL1* showed markedly high expression in stems of both *B. napus* and its two diploid progenitors. Furthermore, the homologous genes of *EIL3* had relatively high expression levels in leaves of *B. napus*, but expressed lower in its two diploid progenitors, which indicated that *EIL3* might play a more important role in leaves of *B. napus* after hybridization and polyploidization.

To investigate whether the expression patterns of all *EIN3/EIL* genes in four tissues changed in the allotetraploid *B. napus* and its two diploid progenitors during evolutionary process, the previously mentioned eleven gene pairs (Table 2) that might have an evolutionary relationship were analyzed for their expression patterns. The FPKM (fragments per kilobase million) values of these gene pairs were shown in Table 5. By comparison, the gene pairs in the C3 sub-clade were highly conserved both in terms of gene structure and gene expression pattern. Specifically, all four gene pairs in the C3 sub-clade had the same gene structure, and the expression patterns of the three gene pairs were consistent. Moreover, there was a gene pair (*BoEIL1* and *BnAEIL1b*) that changed greatly in expression pattern. *BoEIL1* was highly expressed in all four tissues in *B. oleracea*, but *BnAEIL1b* was not expressed in the four tissues in *B. napus*. This suggested that this gene might have undergone functional changes during the process of polyploidization. There was also a significant change in the expression of *BrEIL3a* and *BnAEIL3a* in leaves. *BrEIL3a* was highly expressed in leaves of *B. oleracea*, but *BnAEIL3a* was not expressed in leaves of *B. napus*, indicating that this gene no longer plays an important role in the leaves of *B. napus*.

**Table 5:** The expression patterns of *EIN3/EIL* gene pairs with potential direct evolutionary relationships

Clade	Gene pairs with evolutionary relationship	Flowers_FPKM	Leaves_FPKM	Siliques_FPKM	Stems_FPKM	Gene structure^a^
A1 sub-clade	BoEIL1	28.80	27.85	32.86	50.47	S
	BnAEIL1b	0	0	0	0	
B3 sub-clade	BrEIL3a	11.38	9.53	7.79	6.19	S
	BnAEIL3a	5.37	0.00	4.87	5.02	
	BoEIL3a	19.85	8.06	15.98	14.05	S
	BnCEIL3b	5.36	5.62	5.56	5.16	
	BrEIL3c	0.31	0.04	0.08	0	D
	BnAEIL3d	0.59	0.28	0.48	0.47	
	BoEIL3b	9.75	3.84	6.78	6.23	D
	BnCEIL3c	3.74	4.39	1.93	2.38	
C1 sub-clade	BrEIL2	0.12	0.09	0.29	0.12	D
	BnAEIL2a	0	0	0.06	0.16	
	BoEIL2b	0.02	0	1.30	0.06	D
	BnCEIL2b	0	0	0.02	0.15	
C3 sub-clade	BrEIL4a	0	0	0	0	S
	BnAEIL4b	0	0	0	0	
	BoEIL4a	0	0	0.03	0.05	S
	BnCEIL4a	0	0	0	0	
	BrEIL4b	0	0	0	0	S
	BnAEIL4d	0	0	0	0	
	BoEIL4b	0.76	0.13	1.11	0.67	S
	BnCEIL4c	0	0.08	0.22	0	

^a^Gene structure was used to show whether the gene pairs had the same intron/exon structure. S represented they have same gene structure and D represented they have different gene structure

To further explore the bias in expression of *EIN3/EIL* genes in the four tissues of the allotetraploid *B. napus*, we analyzed the expression of *EIL2*, *EIL3* and *EIL4* based on FPKM values. An interesting phenomenon was that the expression of these three genes was biased towards the diploid progenitor *B. rapa* in all four tissues of *B. napus*.

## Discussion

Polyploidization is a common event in the evolutionary history of various species [41], and polyploidy is prevalent in plants, especially in angiosperms [42]. After polyploidization, plants obtain more than one set of genomes with a series of genomic changes other than a simple addition. The group of *B. napus* and its diploid progenitors (*B. rapa* and *B. oleracea*) is applicable for studying the polyploidization. Moreover, the *EIN3/EIL* gene family is an important gene family and *EIN3/EIL* genes affect the growth and development of plants by participating in the ethylene signal transduction process [10, 11]. Previous studies on the *EIN3/EIL* gene family have been conducted in poplar and Rosaceae plants [27, 43], whereas there have been no reports on this family in *Brassica*. Therefore, we identified and analyzed the *EIN3/EIL* gene family in the allotetraploid *B. napus* and its diploid progenitors to insight into the evolution of this gene family during the natural formation of *B. napus*.

### *EIN3/EIL* gene family in *B. napus* acquired introns during polyploidization

Introns are non-coding sequences that interrupt the coding regions of genes in eukaryotes. Moreover, introns are prominent markers of eukaryotic protein-coding genes [44–46] and are critical components for genome adaptation to environmental challenges [47]. In this study, some *EIN3/EIL* genes in *B. napus* acquired introns. Statistical analysis showed that only one (*EIL3*) of the six (16.7%) *EIN3/EIL* genes in Arabidopsis contained an intron, and the remaining five genes had no introns. 42.9 and 28.6% of *EIN3/EIL* genes contained introns in *B. rapa* and *B. oleracea*, respectively. However, up to 77% of the *EIN3/EIL* genes contained introns in *B. napus*, which might bring some benefits to *B. napus*. Introns may retain mutational disturbances, thereby buffering the coding exons from mutations and protecting exons to make genes more conserved in evolution [48, 49]. Moreover, the presence of introns has some distinct advantages for organisms [48, 50]. First, introns can increase protein diversity by alternative splicing or exon shunting [51–53]. Second, introns can regulate gene expression [52], and some introns named intron-mediated enhancement (IME) can also promote gene expression [54]. Third, introns can produce non-coding RNAs to participate in some regulatory processes [55]. In addition, introns can increase the function of proteins by obtaining functional domains, thereby increasing the versatility of proteins [49]. Finally, introns play key roles in some biological processes, such as transcriptional coupling, splicing and mRNA export [56]. Of course, the relatively large number of introns in the *EIN3/EIL* gene family of *B. napus* might bring these advantages to the organism, but this hypothesis needs further study.

### Homolog expression of *EIN3/EIL* genes in *B. napus* is biased towards its diploid progenitor *B. rapa*

*B. napus*, a young allotetraploid, formed only ~ 7500 years ago by the natural hybridization and polyploidization of *B. rapa* and *B. oleracea* [22]. Whole-genome sequencing of *B. napus* and its diploid progenitors also provided us with a valuable opportunity to explore how the gene families or sub-genomes were affected in young polyploids. In the current study, there was no large-scale gene loss in the *EIN3/EIL* gene family in *B. napus*. Lower gene loss rates are generally thought to promote the wide spread of polyploids in the early stages of their formation and contribute to their fast diversification [23, 42, 57, 58]. In fact, the chromosomal DNA and gene loss rate can reach 15% during the first generation of some artificial/synthetic tetraploids [59, 60].

The homolog expression of *EIN3/EIL* genes in *B. napus* was biased towards its diploid progenitor *B. rapa*. On the one hand, the distribution of *EIN3/EIL* genes on the A genome in *B. rapa* and the A sub-genome in *B. napus* was identical, except for the A_r_07-A_n_07 chromosomes (Fig. 1). Only a few *EIN3/EIL* genes maintained their number and relative position on the C genome in *B. oleracea* and the C sub-genome in *B. napus*. On the other hand, the expression bias analysis showed that all three genes that could be analyzed (*EIL2*, *EIL3* and *EIL4*) were biased towards *B. rapa* in the four tissues (leaves, stems, flowers and siliques).

### The promoter of *EIN3/EIL* genes in *B. napus* contains more *cis*-acting elements than its diploid progenitors

*Cis*-acting elements of the gene promoter regions control the gene responses in the organism and constitute the basic functional link between the complex regulatory networks of genes [61]. *Cis*-acting elements involve extensive biological functions, such as plant growth and development and hormone responses. Different genes have various classes of *cis*-acting elements to exert different biological functions. The *EIN3/EIL* gene promoter region is rich in *cis*-acting elements in poplar, and two of them (CAAT-box and TATA-box) are present in all *EIL* genes [43]. These two elements are common *cis*-acting elements in the promoter region of eukaryotic genes, where the CAAT-box forms the binding site for RNA transcription factors and regulates the frequency of gene expression [62], and another TATA-box contains the binding site of general transcription factors or histones and involved in the transcription process along with its binding factor [63]. In this study, these two *cis*-acting elements were also present in all *EIN3/EIL* gene promoters in *B. napus* and its diploid progenitors. In addition, as shown in Fig. 6, the *cis*-acting elements of *EIL3/EIL* gene promoters were divided into three categories (plant development and growth, phytohormone responses and light responsiveness) according to the biological processes. Interestingly, the total number of *cis*-acting elements in the *EIN3/EIL* gene promoters of *B. napus* (373) was far more than the sum of the elements in its diploid progenitors (235). Further analysis revealed that the number of elements involved in the phytohormone responses in *EIN3/EIL* gene promoters of *B. napus* (99) was similar to the sum of the elements in its diploid progenitors (95). Therefore, the quantitative difference mainly exists in *cis*-acting elements related to plant development and growth and light responsiveness. Furthermore, the total number of *cis*-elements involved in plant development and growth in the *EIN3/EIL* promoters of *B. napus* was 2.9 times that in the diploid progenitors, and the number of light responsiveness elements was 1.8 times that in the diploid progenitors. Two *cis*-elements showed significant differences, namely skn_1 motif and Box I. Specifically, there were 34 skn_1 motifs and 16 Box I in the *EIN3/EIL* gene promoters of *B. napus*, but there were only 4 skn_1 motifs and 1 Box I in the two diploid progenitors. The skn_1 motif is a *cis*-acting regulatory element required for endosperm gene expression, and Box I is a light-responsive element. Therefore, the increased number of *cis*-elements in *EIN3/EIL* gene promoters of *B. napus* might enhance their functions in endosperm gene expression and light responsiveness.

## Conclusions

In this study, 13, 7 and 7 *EIN3/EIL* genes were identified in allotetraploid *B. napus*, the A_n_ genome donor *B. rapa* and the C_n_ genome donor *B. oleracea*, respectively. After analysis, many members of *EIN3/EIL* gene family in *B. napus* acquired introns during polyploidization, which might bring some advantages to the organism. Moreover, the *EIN3/EIL* genes in *B. napus* is biased towards its diploid progenitor *B. rapa* rather than *B. oleracea*, from the two aspects of gene localization and gene expression. In addition, the promoter of *EIN3/EIL* genes in *B. napus* contains more *cis*-acting elements than its diploid progenitors, which might enhance their functions in endosperm gene expression and light responsiveness. In short, our results indicated allotetraploid *B. napus* might have potential advantages in some biological aspects, and these results can increase the understanding of the evolution of the *EIN3/EIL* gene family in *B. napus*, therefore provided more reference for future research about polyploidization.

## Methods

### Plant materials

The seeds of the tetraploid *B. napus* (cv. Darmor) and its diploid progenitors *B. rapa* (cv. Chiifu) and *B. oleracea* (cv. Jinzaosheng) were obtained from the Oil Crops Research Institute, Chinese Academy of Agricultural Sciences, China. These materials were grown under natural conditions in Wuhan, China, and inflorescences were bagged to prevent pollen contamination before blossom. Young leaves, inflorescence stems, blooming flowers and siliques (10DAP, Days after Pollination) of 6-months materials were simultaneously and quickly frozen in liquid nitrogen for later use.

### Identification of *EIN3/EIL* genes

The genome data of *B. napus* and its two diploid progenitors, *B. rapa* and *B. oleracea*, were obtained from the BRAD database (http://brassicadB.org/brad/) [64]. Six EIN3/EIL protein sequences from *A. thaliana*, acquired from the TAIR database (http://www.arabidopsis.org/), were used as queries to perform BLASTp searches (E-value <1e-5) with all proteins from these three species. To identify the *EIN3/EIL* genes in three *Brassica* genomes accurately, all putative protein sequences were confirmed by searching for the EIN3/EIL domain (pfam04873) using CD-search in the NCBI Conserved Domain Database (CDD; https://www.ncbi.nlm.nih.gov/cdd) [65]. In this study, only proteins containing the complete EIN3/EIL domain were considered EIN3/EIL proteins. Finally, the identified *EIN3/EIL* genes were manually named according to their homologous relationships with the *EIN3/EIL* genes in *A. thaliana*. EIN3/EILs in *Oryza sativa*, *Zea mays*, *Gossypium raimondii* and *Populus trichocarpa* were identified using the same methods as described above, and the genome data of all these species were obtained from the Phytozome database (https://phytozome.jgi.doe.gov/pz/portal.html).

### Chromosome location and gene structure analysis

The location information of *EIN3/EIL* genes in *B. napus* and its two diploid progenitors was collected from the BRAD database, and their physical positions were drafted to the corresponding chromosomes by the software MapInspector. The exon/intron structures of *EIN3/EIL* genes were analyzed using Gene Structure Display Server (GSDS) 2.0 (http://gsds.cbi.pku.edu.cn//index.php) [66].

### Conserved motif and characteristic analysis

Conserved motifs in EIN3/EIL proteins were investigated by online MEME server (http://meme-suite.org/tools/meme) [67], with the max motif number as 10 and the other parameters as default values. Moreover, the physico-chemical characteristics of EIN3/EIL proteins in *B. napus* and its two diploid progenitors were calculated by the online ProtParam tool of ExPASy (http://weB.expasy.org/protparam/) [68], including sequence length, molecular weight (MW), theoretical isoelectric point (pI), instability index (II), aliphatic index and grand average of hydropathicity (GRAVY). The tertiary structure of EIN3/EIL proteins in *B. napus* and its diploid progenitors was predicted using the homology modeling method (SWISS-MODEL, https://www.swissmodel.expasy.org).

### Phylogenetic relationship analysis

The EIN3/EIL protein sequences in 6 dicots (*B. rapa*, *B. oleracea*, *B. napus*, *A. thaliana*, *Gossypium raimondii* and *Populus trichocarpa*) and 2 monocots (*Oryza sativa* and *Zea mays*) were aligned using ClustalX. Subsequently, phylogenetic relationships were presumed by analyzing a Maximum Likelihood (ML) tree that was constructed by MEGA 7.0.26 [69] with 1000 bootstrap replicates. Finally, the online Interactive Tree of Life (iTOL, http://itol.embl.de/) [70] was used to decorate this phylogenetic tree.

### Gene duplication and syntenic analysis

Duplicated *EIN3/EIL* genes were identified by BLASTn using their coding sequences (CDSs). The two criteria were (a) coverage of sequence length > 80% and (b) identity of aligned regions > 80% [71]. DnaSP software (version 5.10.01) was used to calculate the synonymous (Ks) and nonsynonymous (Ka) substitution rates of duplicated *EIN3/EIL* gene pairs [72]. Then, evolutionary constraint (Ka/Ks) was calculated to analyze the selective pressure. The syntenic genes of *EIN3/EILs* in *B. napus* and its two diploid progenitors were found in the BRAD database, and Circos software was applied to express the syntenic relationship between them [73].

### Promoter sequences and gene expression analysis

The promoter sequences, which were the 1500 bp upstream of the transcription start site (TSS) of the *EIN3/EIL* genes, were acquired from the BRAD database, and the *cis*-elements in the promoters were analyzed using the Plant *Cis*-Acting Regulatory Element (PlantCARE) server (http://bioinformatics.psb.ugent.be/webtools/plantcare/html/) [32]. Plant materials were collected for transcriptome sequencing on the Illumina HiSeq X-Ten platform. To determine the expression patterns of *EIN3/EIL* genes in *B. napus* and its two diploid progenitors, RNA-seq data of four major tissues (stems, leaves, flowers and siliques) were analyzed. FPKM values were used to represent the gene expression levels. FPKM values were normalized by Z-values, and Z-values were calculated by the following formula. $\mathrm{Z}-\mathrm{value}=\frac{\log 2\left(\mathrm{FPKM}\right)-\mathrm{Mean}\ \left(\log 2\left(\mathrm{FPKM}\right)\ \mathrm{of}\ \mathrm{all}\ \mathrm{samples}\right)}{\mathrm{standard}\ \mathrm{deviation}\ \left(\log 2\left(\mathrm{FPKM}\right)\ \mathrm{of}\ \mathrm{all}\ \mathrm{samples}\right)}$ . The heatmap of gene expression was generated using Multi Experiment Viewer (MeV; version 4.9.0) software.

## Additional files

Additional file 1: **Figure S1.** The predicted tertiary structure of all EIN3/EIL proteins in *B. napus* and its diploid progenitors. The predicted structure of BnCEIN3 (**A**). The predicted structure of BnAEIL1b, BnCEIL1a, BoEIL1 and BrEIL1 (**B**). The predicted structure of BnCEIL2b and BoEIL2b (**C**). The predicted structure of BnAEIL2a (**D**). The predicted structure of BoEIL2a (**E**). The predicted structure of BrEIL2 (**F**). The predicted structure of BnAEIL3d, BoEIL3b and BrEIL3c (**G**). The predicted structure of BnCEIL3b and BoEIL3a (**H**). The predicted structure of BnCEIL3c (**I**). The predicted structure of BrEIL3b (**J**). The predicted structure of BnAEIL4b, BnCEIL4a and BoEIL4a (**K**). The predicted structure of BnAEIL4d and BrEIL4b (**L**). The predicted structure of BoEIL4b (**M**). The predicted structure of BnAEIL3a and BrEIL3a (**N**). The predicted structure of BnCEIL4c (**O**). The predicted structure of BrEIL4a (**P**). (TIF 6803 kb)

Additional file 2: **Table S1.** The FPKM values of identified *EIN3/EIL* genes in four major tissues of *B. napus* and its diploid progenitors (*B. rapa* and *B. oleracea*). (XLSX 15 kb)

## Abbreviations

*A. thaliana*: *Arabidopsis thaliana*
AR: Acidic region
*B. napus*: *Brassica napus*
*B. oleracea*: *Brassica oleracea*
*B. rapa*: *Brassica rapa*
BLAST: Basic Local Alignment Search Tool
BRI-V: Basic regions I-V
DBD: DNA-binding domain
*EIN3/EIL*: *Ethylene-insensitive 3/ethylene-insensitive 3-like*
ERF1: ETHYLENE-RESPONSE-FACTOR1
ET: Ethylene
FPKM: Fragments per kilobase million
GRAVY: Grand average of hydropathicity
II: Instability index
IME: Intron-mediated enhancement
Ka: Non-synonymous substitution
Ks: Synonymous substitution
ML: Maximum Likelihood
MW: Molecular weight
PR: Proline-rich region
Q/D: Asp/Gln
TSS: Transcription start site
