# Supplementary figures and images for: Genome-wide identification and analysis of the EIN3/EIL gene family in allotetraploid Brassica napus reveal its potential advantages during polyploidization
Source: BMC Plant Biol. 2019 Mar 21;19:110. doi: 10.1186/s12870-019-1716-z (PMC6429743; doi:10.1186/s12870-019-1716-z)

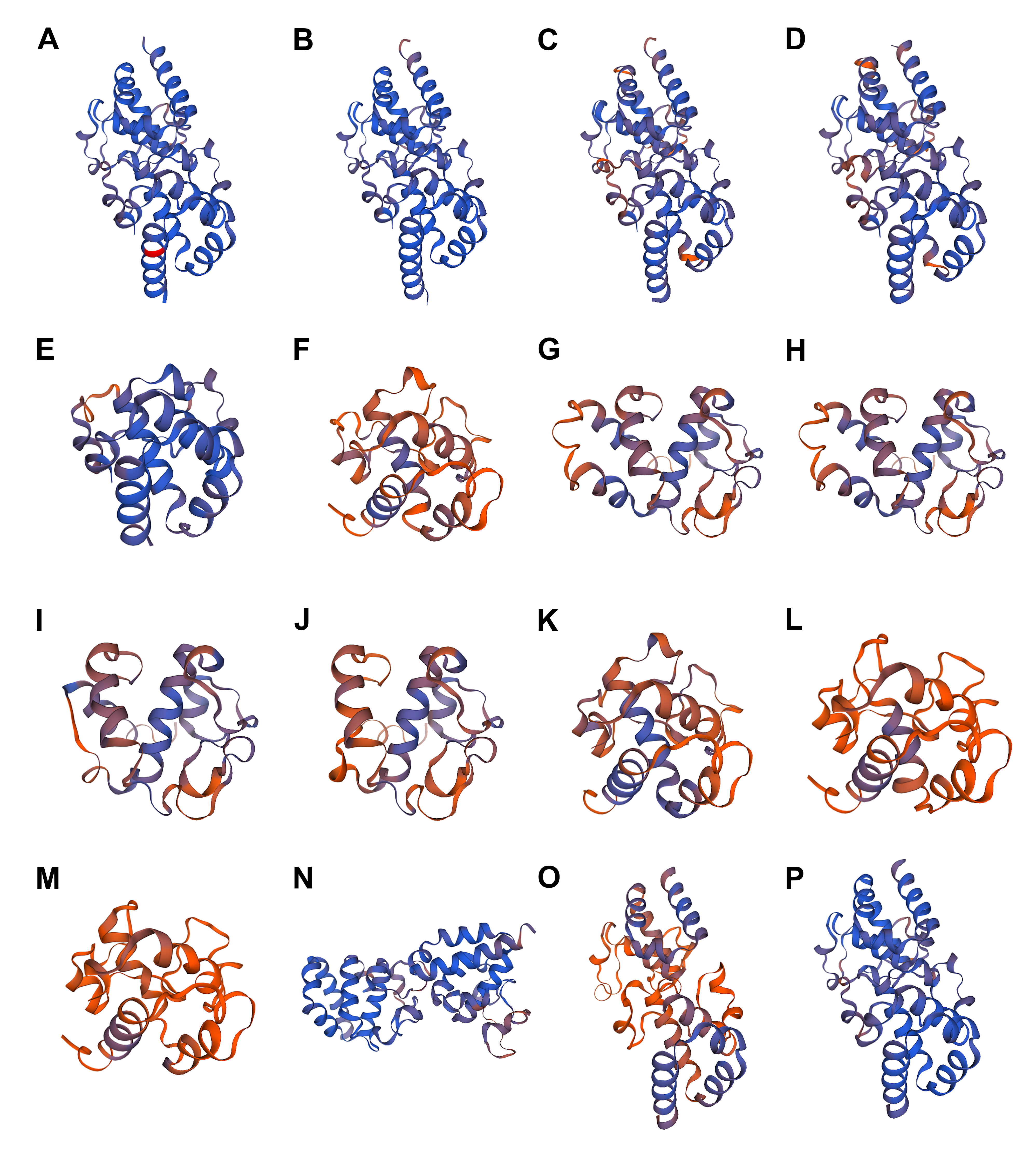

Supplement: Supplementary file 1 — Figure S1. The predicted tertiary structure of all EIN3/EIL proteins in B. napus and its diploid progenitors. The predicted structure of BnCEIN3 (A). The predicted structure of BnAEIL1b, BnCEIL1a, BoEIL1 and BrEIL1 (B). The predicted structure of BnCEIL2b and BoEIL2b (C). The predicted structure of BnAEIL2a (D). The predicted structure of BoEIL2a (E). The predicted structure of BrEIL2 (F). The predicted structure of BnAEIL3d, BoEIL3b and BrEIL3c (G). The predicted structure of BnCEIL3b and BoEIL3a (H). The predicted structure of BnCEIL3c (I). The predicted structure of BrEIL3b (J). The predicted structure of BnAEIL4b, BnCEIL4a and BoEIL4a (K). The predicted structure of BnAEIL4d and BrEIL4b (L). The predicted structure of BoEIL4b (M). The predicted structure of BnAEIL3a and BrEIL3a (N). The predicted structure of BnCEIL4c (O). The predicted structure of BrEIL4a (P). (TIF 6803 kb) [file 12870_2019_1716_MOESM1_ESM.tif]
